# Clues to the Evolution of the Malarial Chromosome

**DOI:** 10.1371/journal.pbio.0030361

**Published:** 2005-09-13

Understanding the recombination patterns across a chromosome—determining the positions and frequency of genetic exchanges between homologous chromosomes—is crucial for understanding and tracking inheritance of traits. Mapping genes that affect parasites' traits, such as responses to various antimalarial agents, is possible because, during meiosis, homologous chromosomes line up and may exchange segments. Genes—or any polymorphic bits of DNA—that are close together tend to remain linked during this process, while those far apart tend to become separated. Identifying and following polymorphic markers through multiple generations is a key technique for genetic mapping.

For *Plasmodium falciparum*, the microbe that causes malaria, chromosomal mapping is necessary for understanding the evolution of the parasite and development of drug resistance, but multiple factors make this a complex task. In this issue, Jianbing Mu and colleagues use single nucleotide polymorphisms (SNPs) to evaluate some of these factors, and set the stage for further mapping of this important parasite's genome.

The authors began by locating 183 SNPs spaced across Chromosome 3 in 99 *P. falciparum* populations from throughout the world. Not all SNPs were found in all populations, indicating a more recent evolutionary origin for some SNPs; these differences were then used to track evolution and migration in parasites. Statistical analysis of the SNPs allowed the populations to be parsed into five groups, largely corresponding to continents. More refined analysis of the SNPs revealed possible migratory history, including a recent migration of an African variety to coastal South America.[Fig pbio-0030361-g001]

**Figure pbio-0030361-g001:**
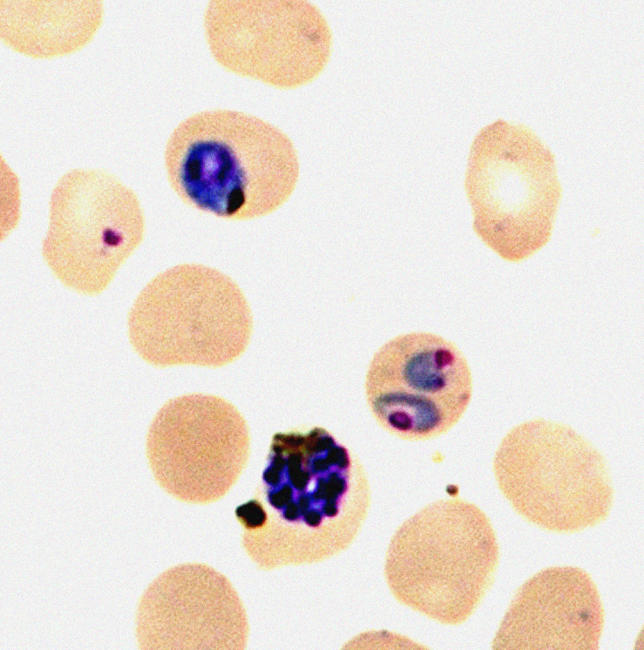
Plasmodium falciparum, the microbe that causes malaria, infects red blood cells. By analyzing different populations of the pathogen from around the world, researchers found clues to its genome structure that will be important for identifying genes that contribute to drug resistance and virulence

Mu and colleagues also showed for the first time that the historical rate of recombination varies widely—over 20-fold—among different populations. A large part of the variation is due to a combination of the frequency of infections with multiple parasite strains (because sexual recombination occurs only within an infected mosquito) and the degree of inbreeding within a parasite population. Inbreeding tends to lower the extent of detectable recombination events, while multiple infections by different strains increase it.

Despite the wide differences in recombination rates, all populations had a similar clustering of recombination “hot spots” at the middle and ends of the chromosome. Recombination is most likely to occur at these spots, and the similar localization reflects either the common evolutionary history of all the populations or localization of crossover events to particular genomic regions.

The authors compared their results from population structure analysis with those using SNPs from genes that might be influenced by drug pressure. Their results showed that misleading inferences about the parasite population structures could be derived using information from genes that are potentially under drug selection.

These results are important because they provide information on the multiple complex factors that must be considered in understanding the genomic structure of *P. falciparum*, which is critical for identifying genes that contribute to phenotypes such as drug resistance and virulence. Reseachers conducting future mapping studies will be able to draw on the important findings and caveats revealed by this work to refine their own methods and interpret their results. —*Richard Robinson*

